# Does Social Media Use Increase Depressive Symptoms? A Reverse Causation Perspective

**DOI:** 10.3389/fpsyt.2021.641934

**Published:** 2021-03-23

**Authors:** Andree Hartanto, Frosch Y. X. Quek, Germaine Y. Q. Tng, Jose C. Yong

**Affiliations:** ^1^Singapore Management University, Singapore, Singapore; ^2^Nanyang Technological University, Singapore, Singapore

**Keywords:** social media, depression, adolescent, reverse causation, screen time

According to the World Health Organization (1), 264 million individuals worldwide suffer from depression—a condition characterized by feelings of low self-worth, impaired concentration, and disturbed sleep, among various other maladaptive symptoms (2). Adolescents between 13 and 18 years of age are also vulnerable (3), with a 52% increase in the prevalence of depression among adolescents from 2005 to 2017 (4). Depression is tied to many serious problems including failure to complete education, higher unplanned parenthood rates, poorer interpersonal relations, and heightened risk of substance abuse and suicidality (5–7).

Researchers seeking to understand this prevalence of depression in modern society have identified social media use as a key risk factor (8, 9). Underscoring the ubiquity of social media in people's lives today, 3.8 billion people in the world are active users of at least one social networking site (10). Cross-sectional and correlational trend analyses appear to show increases in depression rates alongside increased social media engagement [(9, 11–14); also see (15), for a contrasting result]. Although the link between social media use and depression is contentious (16, 17), several have nonetheless implicated social media as pervasive and detrimental to psychological well-being. For instance, some studies have been argued that social media may harm users by exposing them to negative stimuli such as unwholesome content, cyberbullying, unhealthy social comparisons, and feelings of inferiority (18, 19).

However, there are good reasons to suspect that these conclusions are premature or incorrect. For instance, the haste to pass social media a guilty verdict might stem from moral panic brought about by the fear of contemporary technology (20), where the third-person effect in which individuals overattribute certain behaviors to the influence of mass communication (21) is a possible catalyst. Having such a convenient culprit for depression seriously oversimplifies our understanding of the etiology surrounding social media and depression (12).

Several lines of reasoning also suggest that the direction of social media causing depression might instead be reversed. The heavy reliance on correlational data severely limits our ability to infer directionality and argue that exposure to social media causes depression (11, 22). Some theories also indicate that depressive symptoms drive social media use. According to the theory of compensatory internet use (23), people may view online activities such as social media as a means to alleviate negative feelings or fulfill unmet psychosocial needs. For example, an individual suffering from depressive symptoms may turn to social networking sites for social validation through gaining likes and followers. The sociocognitive model of internet addiction (24) similarly proposes that because online and social media stimuli can be psychologically rewarding, people are incentivized to stay engaged, but those with poorer self-regulation (e.g., individuals with psychological disorders) are especially susceptible to developing harmful social media habits. The tendency for social media use to be precipitated by psychosocial problems like low self-esteem, insecurity, and depression also makes sense from an evolutionary perspective. As highly social creatures, humans evolved a desire for belonging and social acceptance (25), which is facilitated by social monitoring motives to detect cues for social approval or rejection (26). Individuals who are insecure about being accepted are motivated to seek information that may reaffirm their social status, leading to an obsession with social comparisons and other social diagnostic cues (27). Thus, the evolutionary perspective suggests that social media can hijack our evolved tendency to monitor for social cues and perceive social information as rewarding (28), and depressed individuals are especially vulnerable to being sucked in by social media as they experience a stronger need to alleviate feelings of insecurity, low self-worth, or hopelessness. These arguments from multiple perspectives provide compelling justification to take the reverse association more seriously.

## Evidence for Reverse Causality in the Relationship between Social Media and Depressive Symptoms

Considering that increased social media use may be an outcome of depressiveness rather than an antecedent, what does the available data show? Here, we critically examine the literature and consider the evidence from longitudinal and experimental studies.

### Longitudinal Findings

While longitudinal designs alone do not provide grounds for causal conclusions, they at least allow the temporal ordering of key variables to be determined (29). Several recent longitudinal studies show null or even disconfirming evidence for social media as a precedent of depression. For instance, Jensen et al. (30) found that baseline frequency of social media use did not predict increased daily depressive symptoms at 1- to 2-year follow-up in a demographically representative sample of adolescents. More prolonged studies spanning 8 or 9 years have further corroborated the view that frequency of social media use does not longitudinally predict depressive symptoms in adolescents even when studied across developmental periods from adolescence to young adulthood (31, 32). Further, using a longitudinal panel design on a sample of American adults, Hampton (33) showed that social media use was in fact associated with *reduced* psychological distress due to the social opportunities afforded by social networking sites.

Some recent longitudinal studies have also shown that depressive symptoms may precede escalated social media use. For instance, Heffer et al. (34) followed samples of adolescents and undergraduates over 2 and 6 years, respectively, and demonstrated that depressive symptoms preceded increased social media use among female adolescents, while social media usage did not precede depressive symptoms across both samples. Similarly, Raudsepp and Kais's (35) 2-year study of adolescent girls revealed that baseline levels of depressive symptoms predicted problematic social media use, whereas prior social media use did not predict depressive symptoms. Examining the within-person effects of depressive symptoms on increased social media use over 6 years during early and late adolescence, Puukko et al. (36) found that depressive symptoms predicted increases in social media use for both boys and girls. Conversely, social media use was not found to predict depressive symptoms.

A bidirectional relationship may also exist due to a cascading feedback loop whereby depression engenders compensatory social media use, which in turn aggravates depressive symptoms due to unhealthy social comparisons and other detrimental forms of social interaction (37, 38). Raudsepp (39) pointed to this bidirectionality by showing that problematic social media use (i.e., using social media in an unhealthy or maladaptive manner) predicted depressive symptoms over a span of 2 years. Thus, researchers who focus only on the depressive effect of social media use may neglect important predisposing factors that cause certain individuals to use social media problematically. Together, the longitudinal evidence suggests that claims that exposure to social media leads to depressive symptoms might be exaggerated, and the reverse relationship whereby depressiveness prompts more (and unhealthy) social media use has been overlooked.

### Experimental Findings

Although longitudinal designs can ascertain temporal ordering, they do not rule out confounding or third variables that may influence or obscure the relationship between depressive symptoms and social media engagement. Meta-analyses of cross-sectional studies on social media use and depression also report small pooled effect sizes (9, 11, 12), suggesting the presence of extraneous variables. To address issues of causality and control, we consider experimental research on the social media-depression link.

Most if not all experimental studies of this relationship have manipulated social media use to isolate their effects on depression-related outcomes, probably because (1) this is easier to do than the reverse—manipulating participants' depressiveness and observing their social media behavior, and (2) the approach of blaming social media dominates over other theoretical approaches. However, experimental results do not support the view that social media use increases depression-related outcomes. For instance, Hall et al. (40) manipulated social media use by randomly assigning undergraduates to varying durations of abstinence (0, 2, 3, and 4 weeks) and found that social media abstention did not account for changes in loneliness or subjective well-being [see (41) for similar finding]. Moreover, Vally and D'Souza (42) showed that a week-long abstinence from social media led to reduced life satisfaction and increased negative affect and perceived stress.

Other experiments suggest that regulated usage rather than complete abstinence may be key to managing the psychological effects of social media. For example, Hunt et al. (43) demonstrated that limiting social media use to 30 min daily alleviated loneliness and depressive symptoms over a span of 3 weeks. Pointing to the modulating role of individual differences, Turel et al. (44) found that a week of social media abstinence reduced perceived stress levels, but only for excessive social media users. This result highlights the importance of accounting for antecedents of problematic social media use, such as depressive tendencies or other predispositions, otherwise psychological outcomes as a function of social media use remains unclear.

## Discussion and Future Directions

The modern pervasiveness of social media use has prompted considerable research effort to understand its psychological repercussions. Much of this work is lamentably correlational, but despite the inability to deduce causality from such data, many researchers have proceeded to point at social media for today's increased rates of depression while ignoring the reverse possibility that depressiveness may, in fact, explain increased social media use. As we described, longitudinal and experimental methods that allow us to probe temporality and causality reveal not so much that social media triggers depressive symptoms, but more so that (1) initial levels of depression-related symptoms predict prospective social media use, and (2) social media engagement that is already problematic (i.e., unhealthy usage caused by some other antecedent factor) exacerbates depression-related outcomes.

To achieve a fuller picture of the social media-depression link as well as a better understanding of how to combat depression, research needs to move toward a deeper analysis of the factors underlying problematic social media activity, such as risk factors for depression that lead to unhealthy social media use. Some considerations include the tendency for individuals with certain predispositions (e.g., depression, low self-esteem, perfectionism) to view social media as a means to reaffirm their self-worth through social comparisons and seeking social validation (23). More severely depressed or stressed individuals may also view social media as a means of escape and spend excessive periods of time aimlessly browsing social networking sites (45). It is thus necessary to understand the factors that push individuals toward unhealthy social media behavior, in which social media use is more a symptom than a cause.

Although emerging research hints at the role of depression in maladaptive social media use, the available data is meager given the lack of studies guided by the reverse causal direction. Aside from broadly calling for more tests of the effects of depressive symptoms on social media use, we also offer several specific suggestions for further research. First, longitudinal studies should go beyond general measures of social media use (e.g., frequency) and examine more fine-grained, qualitative features of social media participation, such as purposes of use, active vs. passive engagement, and degree of addiction or problematic use, which have exhibited asynchronous associations with depressive symptoms (46). For instance, depressive mood has been found to be associated less with active social media use (e.g., commenting, messaging) and more with passive social media use [e.g., scrolling; (47)]. Second, although retrospective self-reports of adolescents' social media use are most commonly employed, they may be biased and suffer from underestimation (48). Hence, future longitudinal work should employ objective data logs or screen-time apps as measures of social media frequency (49). Third, longitudinal studies should follow participants over a longer time span and incorporate multiple time points (50) to provide a sufficient window for substantive changes in social media use. Fourth, experimental studies manipulating social media abstention should include an equivalent “placebo abstinence” condition to adjust for extraneous confounds, such as expectancies and loss of personal control (51). Lastly, given the considerable implications of findings from this research direction for adolescents' technology-use regulations and mental health, it is crucial that scholars embrace open scientific practices, such as pre-registration and utilization of the open science framework, to encourage future replications and rigorous reviews that can advance our understanding of how social media use and depressive symptoms are connected (52).

In conclusion, the current paper argued that the reverse causal view that depressiveness drives social media use has been neglected in current research. By emphasizing the need for longitudinal and experimental approaches to ascertain directionality, a better grasp of the dynamics that govern depressive symptoms and maladaptive social media use can be attained.

## Author Contributions

AH conceptualized the manuscript. AH, FQ, GT, and JY wrote the manuscript and contributed to critical revision of the manuscript. All authors contributed to the article and approved the submitted version.

## Conflict of Interest

The authors declare that the research was conducted in the absence of any commercial or financial relationships that could be construed as a potential conflict of interest.
